# The Effect of Platelet-Rich Fibrin and Platelet-Rich Plasma in Secondary Alveolar Bone Grafting in Cleft Lip and Palate Patients: A Systematic Review

**DOI:** 10.3390/jcm13071875

**Published:** 2024-03-24

**Authors:** Showbanaa Thangarajah, Rifqah Nordin, Huann Lan Tan, Hui Yuh Soh, Syed Nabil

**Affiliations:** Department of Oral and Maxillofacial Surgery, Faculty of Dentistry, National University of Malaysia, Kuala Lumpur 50300, Malaysia; p106577@siswa.ukm.edu.my (S.T.); rifqahnordin@ukm.edu.my (R.N.); tanhuannlan@ukm.edu.my (H.L.T.); sohhuiyuh@ppukm.ukm.edu.my (H.Y.S.)

**Keywords:** platelet-rich fibrin, platelet-rich plasma, secondary alveolar bone grating, cleft lip and palate, systematic review

## Abstract

(1) **Background**: Cleft lip, alveolus, and palate are the most common congenital abnormalities in the world, occurring in one in seven hundred live births. Secondary alveolar bone grafting (SABG) is usually performed when the permanent canine root shows one-half to two-thirds of root development. To improve the surgical outcome, supplemental grafting materials such as platelet-rich fibrin (PRF) and platelet-rich plasma (PRP) have been used as an adjunct. This review is designed to assess the efficacy of PRF and PRP in improving the outcome of SABG. (2) **Methods**: A comprehensive literature search was performed until 13 October 2022 on MEDLINE, EMBASE, The Cochrane Library, and Pubmed. The full text of potentially relevant studies was reviewed, and only randomised clinical trials (RCTs) were included based on the inclusion criteria. (3) **Results**: A total of 656 studies were screened, of which four were included for final review. All of the four included studies that evaluated the quantitative or qualitative surgical outcome in varied ways. (4) **Conclusions**: Results of this review suggest that both PRF or PRP and control group (without the use of PRF/PRP) achieved similar successful outcomes in bone height, bone density, and bone volume in both qualitative and quantitative assessment.

## 1. Introduction

Clefts are the most common congenital abnormalities of the head and neck, occurring in 1 in 700 live births [1]. The presence of a cleft brings a child into a journey of multispecialty treatment from birth until early adulthood or even beyond. The restoration and rehabilitation of lip, teeth, and jaw function and morphology is of uttermost importance in cleft lip and palate patients. The very first surgery that a cleft lip and palate patient undergoes is lip repair and this is followed by cleft palate repair by the age of one [2]. Apart from that, as a patient grows, the need to maintain the integrity of the alveolar arch becomes a challenge to clinicians. Thus, one of the main goals for the reconstruction in cleft lip and palate patients is to augment bone in the cleft area [3]. This aims to achieve maxillary arch continuity and provide adequate bone support for the teeth adjacent to the cleft, allow the eruption of the teeth in the cleft area (canine or lateral incisors), aid in orthodontic movement in aligning the permanent dentition, allow for dental implant placements, speech improvements, closure of oronasal fistula, support the alar base and lip, enhance nasal symmetry, and establish good soft tissue contour with adequate keratinised gingiva for periodontal health [4,5]. The alveolar bone grafting (ABG) or augmentation can be divided into primary and secondary. Primary ABG is performed during infancy usually below the age of two following the lip repair, but before or during the palate repair. Secondary alveolar bone grafting (SABG) implies any bone grafting procedure after palatoplasty. SABG is conventionally performed when one-half to two-thirds of the cleft-side permanent canine root has formed but other variants such as early and late SABG are also being practiced. Early SABG is performed between the age of two to five years prior to the eruption of the permanent incisors, while late SABG is after the age of twelve mainly to facilitate orthodontic treatment. Earlier practice of performing primary ABG has generally been abandoned due to the negative effect of the maxillary growth associated with the early intervention [6]. SABG, therefore, is preferred due to its minimal influence on maxillary growth, as most of the maxillary growth is completed by age six to seven years old. Most recent discussion revolves around the advantages of early SABG versus the conventional timing, with the proponents of early SABG suggesting that grafting before the eruption of lateral incisors provides an improved bone volume at the cleft site but without the deleterious effect on maxillary growth [7,8]. 

Over the years, there were various grafting materials and techniques used to reconstruct the alveolar cleft. However, iliac crest bone grafting before the eruption of the permanent canine is generally considered as the gold standard for alveolar cleft reconstruction. Iliac is the preferred donor site due to the plentiful supply of bone in that region, the ease of harvesting, and the convenience of simultaneous harvesting with alveolar preparation [9]. Outside of iliac crest bone grafting, other autogenous bone grafting that has been practiced includes cranium, mandibular symphysis, and tibia. Although autogenous bone grafting has shown promising outcomes, there are cases with a considerable amount of grafted bone resorption or loss [10]. Failure of bone grafting can pose a negative impact to both patients and parents with the need to undergo a second surgery, an additional cost, time consumption, and a delay in treatment rehabilitation planning. Meanwhile, the surgeons need to accommodate for additional operation time, manpower, and hospital stay. In an attempt to reduce unsuccessful outcomes, supplemental grafting materials have been suggested as a positive adjunct for ABG. Grafting material that has been highlighted with capabilities to enhance bone quality and quantity is platelet concentrate, which includes platelet-rich fibrin (PRF) and platelet-rich plasma (PRP) [11,12]. Due to its capability to enhance wound healing and tissue regeneration, it has been applied in a number of oral surgical procedures such as in implantology, exodontia, oroantral communication closure, soft tissue grafting, sinus augmentation, osteonecrosis of jaw, and intracapsular injection [13]. The main advantage of this material is the abundance of growth factors, such as platelet-derived growth factor (PDGF), vascular endothelial growth factor (VEGF), and transforming growth factor beta (TGF-β), which can promote bone growth and reduction in the resorption rate [10]. With the recent increase in interest in using PRF and PRP as an adjunct in various oral grafting procedures, its possible positive effect in a common procedure like SABG is an important subject. The null hypothesis of this study is that there is no positive clinical effect of using PRF or PRP on the outcome of hard tissue regeneration in SABG. This review’s main objective is, therefore, to determine the effect of PRF or PRP on hard tissue regeneration and preservation post SABG. 

## 2. Materials and Methods

This systematic review adhered to a prespecified protocol and the Preferred Reporting Items for Systematic Reviews and Meta-Analyses (PRISMA) statement. This research was carried out systematically to minimise bias and inaccuracy. The PICOS strategy was used for the research question construction: (P) participants: includes studies involving both unilateral or bilateral alveolar cleft patients of 8 to 12 years old; (I) intervention: patients who have undergone SABG with an autogenous graft in combination with PRP or PRF; (C) control: patients who have undergone SABG with autogenous bone grafting without PRP or PRF; (O) outcome: bone grafting surgery quantitative or qualitative outcome assessment. This is assessed by either using 3-dimensional (3D) or by 2-dimensional (2D) methods, which measured the bone volume, bone height, or bone density; (S) study design: only randomised controlled trials (RCT) were included. SABG, for the purpose of this review, is defined as a bone augmentation procedure over the cleft alveolus at the age of 8 to 12 years old. 

Electronic searches of MEDLINE, EMBASE, The Cochrane Library, and PubMed were searched until 13 October 2022 for RCTs related to SABG using a combination of terms: ‘secondary alveolar bone graft’, ‘secondary alveolar bone grafting’, ‘alveolar bone graft’, ‘alveolar bone grafting’, ‘secondary alveoloplasty’, ‘alveolar cleft’, ‘maxillary cleft’, ‘maxillary alveolar cleft’, ‘maxillary alveolus cleft’, ‘maxillary alveoloplasty’, ‘late alveoloplasty’, ‘cleft alveolus’, ‘platelet derivatives’, ‘platelet-rich plasma’, ‘platelet-rich fibrin’, ‘platelet products’, ‘blood products’, ‘blood derivatives’, and ‘autologous blood’ were used (Appendix A). 

Inclusion criteria are studies reporting on the outcome of SABG of unilateral or bilateral cleft alveolus among 1. children of 8 to 12 years old or studies reporting mean age of 8 to 12 years old; 2. no previous history of SABG; 3. RCT studies; 4. articles with full text; 5. articles reporting outcome measurement for the grafting result (Bergland Classification, Chelsea Scale, Bone Density in mean range, Bone Density in Hounsfield Unit (HU), Bone Density in Aluminium Equivalent (Al-Eq), Bone Volume); and 6. a minimum follow up of 3 months after the surgery. For the exclusion criteria, we excluded studies that used anticoagulants, gelling agents, or any other biochemical blood handling for platelet concentrate, secondary data, protocols, pilot studies, case reports, case series, conference proceedings and abstracts, non-English language publications, and studies not involving human subjects. Studies involving syndromic patients and patients who had undergone SABG previously were also excluded. Two reviewers (ST, SN) independently screened the title and abstracts of identified articles following the electronic search. In the next round of assessment, the full texts of selected articles were retrieved and reviewed by the same reviewers to identify eligible papers based on the predetermined inclusion/exclusion criteria. Any differences were resolved through discussion between the reviewers (ST, SN) and disagreements were settled through consultation with one of the authors (RN). 

Critical Appraisal Skills Programme (CASP) tool was used to assess the validity and methodology of the study by two authors (ST, SN) independently. Data were extracted into a standardised data extraction sheet (Microsoft Excel). This included study year published, country conducted, author, study design, number of participants, number of dropouts, number of interventions, study group, control group, cleft types, gender, mean age, PRF production method (protocol of centrifugation), outcome assessment (Bergland Classification, Chelsea Scale, Bone Density (mean), Bone Density (HU), Bone Density (Al-Eq), Bone Volume, and follow-up period. 

## 3. Results

**Figure 1:**
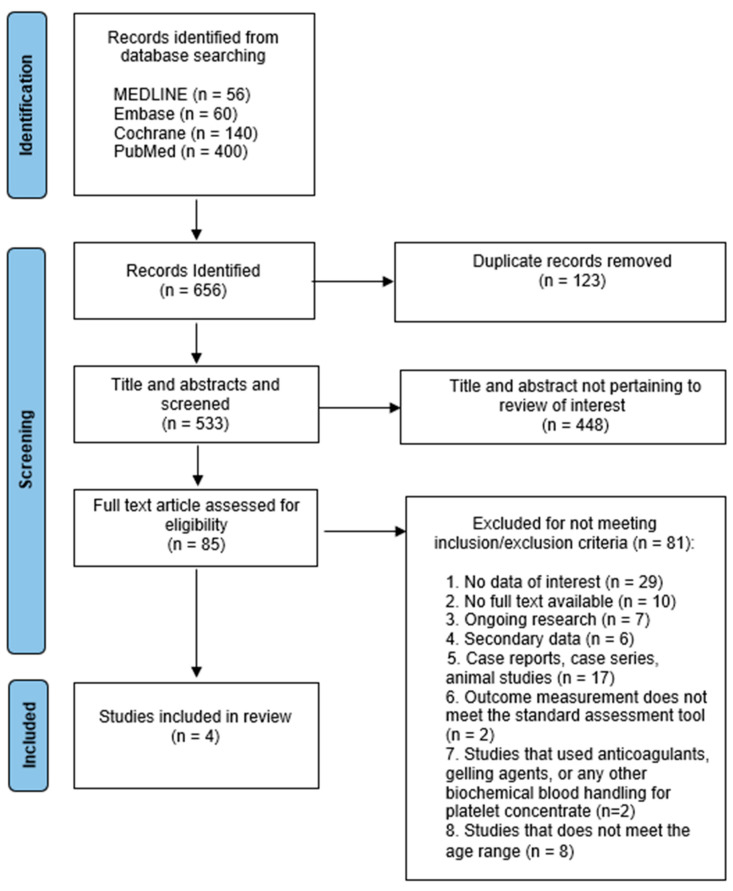
A PRISMA flow chart of the selection and evaluation process of this review.

The electronic database search last updated on 13 October 2022 yielded 400 hits on PubMed, 140 hits on Cochrane, 60 hits on Embase, and 56 hits on MEDLINE, giving a sum of 656 articles from the electronic databases search. Among those articles, 123 duplicates were identified and removed. Following title and abstract screening of the remaining 533 articles, 448 articles were determined as not relevant to this review topic and excluded from the study. Eventually, 85 articles were accepted for full text evaluation. Of these 85 articles, 81 articles did not meet one or more of the pre-determined criteria to be included in this review. A summary of the causes for exclusion is in Figure 1, while the detailed reason for exclusion of each article is listed in Appendix B.

Four RCT articles were accepted for final review (Table 1). They underwent a critical evaluation process using the CASP checklist on quality assessment to obtain the best available valid data for this review. A PRISMA flow chart of the selection and evaluation process is presented in Figure 1. Among the four selected articles, one article reported on quantitative assessment only and three articles reported on both quantitative and qualitative assessment.

### 3.1. Primary Outcome

The bone grafting procedures were measured for quantitative assessment and qualitative assessment outcomes.

#### 3.1.1. Quantitative Assessment

All four studies assessed the outcome of the bone grafting procedure quantitatively. Two studies assessed the grafted site two-dimensionally, one performed three-dimensional assessment, and another one performed both methods to assess the quantitative outcomes (Table 2). 

Only one study showed greater bone retention and reduced resorption rate in two-dimensional quantitative assessment with the use of PRF. Meanwhile, the other three studies on both two-dimensional and three-dimensional reported no significant differences between the control group and study group.

#### 3.1.2. Qualitative Assessment

Three studies assessed the qualitative outcome of the bone grafting procedure. One study assessed the grafted site two-dimensionally, and two did three-dimensional assessment methods to assess the qualitative outcomes (Table 3). 

One study assessing the two-dimensional qualitative outcome with the use of PRP showed significant finding at three months and no significant finding at 12 months, which was consistent with the early remodeling process. The two studies assessing the three-dimensional qualitative assessment do not show any significant differences between the control and study group.

## 4. Discussion

There are several methods to assess the success of SABG. The earlier methods were more focused on the quantity of bones at the grafted site. The initial description was by Bergland et al. (1986) who suggested an assessment on the amount of bone fill by measuring the interdental bone adjacent to the erupted canine [3]. Later, Enmark et al. (1987) introduced an assessment to assess the marginal bone level adjacent to the cleft using intra-oral films on a four-point scale [17]. Next, Kindelan et al. (1997) produced the post-operative bone fill index, which assesses the height of the grafted bone in the cleft on an oblique occlusal in a four-point scale [18]. However, this scale is only applicable for occlusal radiograph. Later, Witherow et al. (2002) introduced the Chelsea Scale, which utilizes intraoral radiographs focusing on the teeth adjacent to the cleft, before the eruption of the canines [19]. This scale involves dividing the tooth on each side of the cleft into four equal parts along the root by bisecting the cleft vertically. The measurement of the bone is then performed in relation to the cleft’s midline, utilising the two neighbouring teeth. Soon after that, a Modified Bergland Score was devised by Hynes and Early (2003), as the original Bergland Score did not consider the basal level of the graft but the height of the interdental bone [20]. These authors proposed the same scoring but including the full height extending from the root apices to interdental height. Moreover, they suggest the score of 3 is sufficient for prosthodontic and periodontal support as well as arch stabilisation in the short term, but is insufficient for patients who require orthognathic surgery. Then, with the advancement of imaging technology, other 3D quantitative and qualitative assessment methods such as bone volume and bone density were introduced. 

Two out of the three papers that reported using 2D quantitative assessment assessed post-grafting bone height measurement using Bergland classification or Chelsea scale. Another one reported using 2D bone resorption rate. Among all three studies that used 2D quantitative assessment, only the study by Dhayashankara et al. found that the PRF group had better bone height as compared to control group [11]. This positive outcome is explained by the ability of platelet-derived products to speed up bone formation and reduce bone resorption in alveolar cleft bone grafting [11,16,21]. However, this study compared the intervention and control group in a purely descriptive manner without any statistical inference analysis [11]. It is unlikely any statistical significance would have been seen with such a small number of sample sizes; thus, their conclusions on the benefits of PRF are questionable. Meanwhile, the study with the Chelsea scale with 2D assessment of resorption rate showed no significant differences between the two groups at 6 months. At 6 months post surgery, the early remodelling phase of the bone is seen where the grafted bone then undergoes a lot of physiological remodelling of the bone grafting [14,22,23]. Although 2D radiographs are still routinely used along with the clinical outcome for bone grafting, the images from these radiographs are unable to provide the volume, morphology, or architecture of the regenerated bone in the cleft defect area [14]. This contributes to the findings that the 2D radiographs can under- or overestimate the bone height up to 17.7% and 21.4%, respectively, when compared with 3D CT scans [24,25]. Furthermore, it has also been reported that the use of a 2D radiograph was not reliable due to distortion of the images at the cleft area [26]. Eventually, many authors have moved on to a 3D imaging method of evaluation to estimate bone loss, although there are still no universally accepted methods to quantify bone grafting outcome [11]. 

Our systematic review had one study assessing quantitative bone volume three-dimensionally (Table 2). Until today, there have been no accepted values in which the amount of grafted bone volume could be defined as “successful” or “failure”. Various papers reported their success rate differently, from 68.4% to 95.0%, probably due to the different definition of success in regard to the achieved bone volume [9]. The study by Thanasut et al. that was included in this study compared the mean volumes, which have no significant statistical differences when compared to the control [14]. If we dissect it further and define the success for bone grafting as gaining a 3D volume of more than 50% and failure as below 50%, the study by Thanasut et al. therefore had a success rate of 62.5% (5/8 had more than 50% bone volume) versus 71. 4% (5/7 had more than 50% bone volume) for the PRF group and the control group, respectively [14]. With the lack of improvement in the use of PRF, Thanasut et al. justify their result by elaborating that autologous iliac crest bone already contains abundant osteoprogenitor cells, which possess higher bone regeneration capacity. Therefore, additional growth factors may not be needed to meaningfully increase bone formation [14]. The same result was shared by another paper by Saruhan et al., which reported no statistical significance difference in the post-operative newly formed bone in PRF group (68.21%) and the control group without PRF (64.62%) [26]. However, the bone volume is also indirectly dependent on the amount of packed bone over the cleft side. Unfortunately, there are no studies reporting on the amount of the bone packed and the cleft volume assessment. The study by Lee et al. reported that the alveolar cleft was measured during operation and an adequate volume of grafted bone was placed in the cleft according to the criteria of Okawachi [16].

In terms of qualitative assessment, all three studies reported on the bone density. Bone density was measured using software assessment, HU unit, and Al-Eq unit. Similarly, there is no standard value to determine the cutoff density to be considered successful bone grafting in any of the methods. All the reported articles performed qualitative assessment between the PRF group and control group [14,15,16]. HU units are commonly used to quantify bone mineral density, where measurements are performed using CBCT [27]. On the other hand, Al-Eq unit is another method used to evaluate bone density by comparing the equivalent thickness of aluminium to standard bone density equipment [28]. The qualitative assessment of this study suggested that PRF may provide higher bone density in a longer post-operative course; however, the resorption rate does not differ significantly in both groups at the end of 12 months [16]. Next, a slight decrease in bone density (*p* < 0.05) could be due to bone fragments being more homogenously amassed within the dense PRF fibrin network, causing the bone density to be diluted by the fibrin [12]. Having said so, PRF is believed to be effective in the first phase of wound healing during the first few weeks after surgery when the growth factors are actively released, reaching its peak of 14 days after surgery, and then it decreases gradually [29]. On the other hand, bone density is believed to be dependent on the remodeling and maturation of the graft, which is demonstrated later after the diminishment of the growth factor [12]. 

All of the included studies in this review assessed the hard tissue outcome of the SABG procedure. Another important aspect in ensuring the success of grafted bone integration is the integrity of soft tissue coverage. Accelerated healing of the enclosing soft tissue would ensure a better outcome of SABG by protecting the grafts from external elements. Accelerated soft tissue healing by PRF or PRP is suggested due to the activation and release of biomolecules such as platelet-specific proteins, platelet-derived growth factor (PDGF), coagulation factors, adhesion molecules, cytokines/chemokines, and angiogenic factors that are capable of stimulating the proliferation and activation of cells involved in wound healing, including fibroblasts, neutrophils, macrophages, and mesenchymal stem cells (MSCs) [30,31]. Its benefits on soft tissue healing are supported by a previous systematic review exploring the effects of PRF on soft tissue wound healing that found positive effects with its use in various tissues including the ear’s auricular, urethra and myocardium, gingiva, oral mucosa, leg ulcers, and others [32]. 

There are several limitations of this review. First, two out of the four studies had a small sample size, making their conclusion on the outcome less assuring. Secondly, the different outcome measures used in each study preclude the possibility of providing a meaningful synthesis of the results. Looking ahead, the future research direction should be on standardisation of PRF processing used in studies, as this may have an effect on the success of the surgery. The first step towards this should be by standardisation of the centrifuging reporting with the disclosure of information such as the rotor dimension, rotor angulation, revolutions per minute (RPM), processing time, composition/size of tubes, and the centrifugation model [33]. Apart from that, a detailed calculation of cleft volume defect size and the amount of bone indicated for harvesting should be standardised prior to surgery to avoid underpacking or overpacking of the bone. Thus, this will give us a guide and a more accurate outcome assessment, which can be standardised. Moreover, there is not a standardised 3D bone volume assessment that is being used, which could aid in the comparative studies. Separately, adequate training in handling PRF should also be taken into consideration, as PRF is technique-sensitive. Consequently, an improper handling of PRF or inexperienced handling could result in biasness of the study. Lastly, the outcome of grafting should also consider the orthodontic treatment that would be received after the surgical intervention, as this could contribute to the increase in the remodeling of the bone. 

## 5. Conclusions

This systematic review found that there is a lack of standardisation in the PRF processing of the methods to assess grafting bone outcome. These diversities make it impossible to compare between studies. Based on the current best available data, this review found that both the PRF group and the control group achieved comparative outcomes in both quantitative and qualitative assessment. 

## Figures and Tables

**Table 1:** Articles included in the final review.

Year	Author	Title	Number of Subjects	Number of Assessed Surgical Site	Age/Mean Age (years)	PRF Production Method (Protocol of Centrifugations)	Quantitative Outcome Assessment	Qualitative Outcome Assessment
2021	Thanasut et al. [14]	Platelet-rich fibrin did not affect autologous bone graft in repairing alveolar clefts	13	15	Study: 9.8 ± 1.6, Control: 10.3 ± 1.9	3000 rpm × 10 min	Bone height (2D), bone volume (3D)	Bone density (3D)
2020	Dhayashankara et al. [11]	A comparative evaluation of iliac crest bone graft with and without injectable and advance platelet-rich fibrin in secondary alveolar bone grafting for cleft alveolus in unilateral cleft lip and palate patients: A randomised prospective study	30	30	Study: 9.7 Control: 8.5 Overall: 9.1	A-PRF 1300 rpm × 8 min, I-PRF 700 rpm × 3 min	Bergland Classification (2D)	-
2018	Omidkhoda et al. [15]	Efficacy of Platelet-Rich Fibrin Combined with Autogenous Bone Graft in the Quality and Quantity of Maxillary Alveolar Cleft Reconstruction	10	10	11.3 ± 0.83	3000 rpm × 10 min	Bone resorption rate (3D)—height and thickness	Bone density Hounsfield unit HU (3D)
2009	Lee et al. [16]	A quantitative radiological assessment of outcomes of autogenous bone graft combined with platelet-rich plasma in the alveolar cleft	60	71	7.4 to 12.3	Not mentioned	Bone resorption rate (2D)	2D Bone density assessment with an aluminum equivalence (Al-Eq) value.

**Table 2:** The quantitative outcome assessment (IOPA = Intraoral Periapical Radiograph, CBCT = Cone Beam Computer Tomography).

Year	Author	Assessment Methods	2D or 3D	Assessment Period	Outcome, *n* (%)		Conclusion
					PRF Group	Control Group	
2020	Dhayashankara et al. [11]	Bergland classification	2D using IOPA	3 months	Type I: 8 (53.3%); Type II: 7 (46.7%)	Type I: 4 (26.7%); Type II: 11 (73.3%)	Study group showed greater bone retention and reduced resorption rate. PRF seems to enhance bone formation and reduces the chances of bone resorption in alveolar clefts when admixed with autologous cancellous bone.
				6 months	Type I: 6 (40%); Type II: 8 (53.3%); Type III: 1 (6.7%)	Type I: 3 (20%); Type II: 6 (40%); Type III: 6 (40%)	
2021	Thanasut et al. [14]	Chealsea scale	2D using IOPA	6 months	A: 5 (62.5%); C: 2 (25.0%); D: 1 (12.5%)	A: 3 (42.9%); C: 4 (57.1%)	There was no significant difference between the two groups. PRF did not affect bone regeneration when repairing alveolar clefts with autologous bone graft.
		Percentage of regenerated bone volume (ratio of the post-operative bone volume and the pre-operative cleft space)	3D using CBCT	6 months	64.9 ± 19.6%	67.0 ± 8.7%	
2018	Omidkhoda et al. [15]	Resorption rate (differences in mean thickness—milimeter)	3D using CBCT	3 months	−4.1	−3.2	There was no significant difference between the two groups. PRF in combination with autogenous bone did not have any significant effect on the bone thickness and height in a three-month period.
		Resorption rate (differences in mean height—milimeter)	3D using CBCT	3 months	−2.8	−3.1	
2009	Lee et al. [16]	Resorption rate (percentage of the changes of vertical height of the bone bridge compared to baseline at 1 week post operatively)	2D using IOPA	3 months	29.9	28.8	There was no significant difference between the two groups. PRP may enhance bone remodelling in the early phase; however, it is insufficient as a countermeasure against bone resorption in the long term.
				6 months	34.4	33.6	
				12 months	32.9	34.9	

**Table 3:** The qualitative outcome assessment (IOPA = Intraoral Periapical Radiograph, CBCT = Cone Beam Computer Tomography).

Year	Author	Assessment Methods	Assessment Period	2D or 3D	Outcome, Mean (mm)		Conclusion
					PRF Group	Control Group	
2018	Omidkhoda et al. [15]	Bone Density (differences in mean HU reading)	3 months	3D using CBCT	−101.3	−88.7	There was no significant difference between the two groups. PRF in combination with autogenous bone did not have any significant effect on the bone density in a three-month period.
2021	Thanasut et al. [14]	Bone Density (differences in mean density assessed with ImageJ software)	6 months	3D using CBCT	0.17 ± 0.15	0.16 ± 0.12	There was no significant difference between the two groups. PRF did not affect bone regeneration when repairing alveolar clefts with autologous bone graft.
2009	Lee et al. [16]	Bone density (percentage in aluminum equivalence (Al-Eq) value compared to value at 1 week post operation as baseline)	3 months	2D using IOPA	79.30%	85.20%	Al-Eq in the PRP group was significantly smaller than that in the non-PRP group at 3 months (*p* = 0.003), and was greater at 12 months (*p* = 0.054). PRP may enhance bone remodelling in the early phase, however, is insufficient as a countermeasure against bone resorption in the long term.
			12 months		93.50%	90.0%	

## Data Availability

The raw data supporting the conclusions of this article will be made available by the authors on request.

## Appendix A

Details on the search strategy used for the electronic search.

## Appendix B

List of 81 articles that did not meet one or more of the inclusion criteria with the detailed reason for exclusion.
